# UltraStrain: An NGS-Based Ultra Sensitive Strain Typing Method for *Salmonella enterica*

**DOI:** 10.3389/fgene.2019.00276

**Published:** 2019-04-03

**Authors:** Wenxian Yang, Lihong Huang, Chong Shi, Liansheng Wang, Rongshan Yu

**Affiliations:** ^1^Aginome-XMU Joint Lab, Xiamen University, Xiamen, China; ^2^School of Information Science and Engineering, Xiamen University, Xiamen, China

**Keywords:** metagenomes, next-generation sequencing (NGS), whole genome sequencing (WGS), *Salmonella enterica*, strain typing

## Abstract

In the last few years, advances in next-generation sequencing (NGS) technology for whole genome sequencing (WGS) of foodborne pathogens have provided drastic improvements in food pathogen outbreak surveillance. WGS of foodborne pathogen enables identification of pathogens from food or environmental samples, including difficult-to-detect pathogens in culture-negative infections. Compared to traditional low-resolution methods such as the pulsed-field gel electrophoresis (PFGE), WGS provides advantages to differentiate even closely related strains of the same species, thus enables rapid identification of food-source associated with pathogen outbreak events for a fast mitigation plan. In this paper, we present UltraStrain, which is a fast and ultra sensitive pathogen detection and strain typing method for *Salmonella enterica* (*S. enterica*) based on WGS data analysis. In the proposed method, a noise filtering step is first performed where the raw sequencing data are mapped to a synthetic species-specific reference genome generated from *S. enterica* specific marker sequences to avoid potential interference from closely related species for low spike samples. After that, a statistical learning based method is used to identify candidate strains, from a database of known *S. enterica* strains, that best explain the retained *S. enterica* specific reads.Finally, a refinement step is further performed by mapping all the reads before filtering onto the identified top candidate strains, and recalculating the probability of presence for each candidate strain. Experiment results using both synthetic and real sequencing data show that the proposed method is able to identify the correct *S. enterica* strains from low-spike samples, and outperforms several existing strain-typing methods in terms of sensitivity and accuracy.

## 1. Introduction

Rapid pathogen identification is one of the most important issues for microbial community studies for infectious diseases and food security. It is reported that in the United States alone, at each year 31 major pathogens cause 9.4 million episodes of foodborne illness, resulting in 55,961 hospitalizations and 1,351 deaths (Scallan et al., 2011). Foodborne illness poses a $77.7 billion economic burden in the United States annually, excluding indirect costs to the food industry such as reduced consumer confidence, recall losses, or litigation (Mandernach et al., 2013). The faster the sources linked with the outbreak being investigated are identified, the faster the outbreak can be stopped, limiting the potential loss it may cause.

A large number of laboratory (*in vitro*) tools have been developed over the past decades for pathogen identification to assist the diagnosis, treatment, and monitoring of infectious diseases. Traditionally, *in vitro* diagnostics of infectious diseases have been performed using culture-based testing, which usually yields diagnostic results in days. In addition, cultivation of bacteria is not always successful under laboratory conditions due to possibly unsuitable methods. In recent years, deoxyribonucleic acid (DNA) and ribonucleic acid (RNA) based molecular assays (Barghouthi, 2011) have become more routine. A DNA-based *in vitro* assay may take the form of a quantitative or qualitative polymerase chain reaction (PCR) assay where the target for detection is a pathogen-specific gene or an anti-microbial resistance marker. The most common bacterial broad-range PCR methods use primers that recognize conserved DNA sequences of bacterial genes that encode ribosomal RNA (rRNA 16S or 23S) (Greisen et al., 1994). Such methods allow the detection of multiple targets in a single experiment and are faster and more sensitive than culture-based methods. However, these targeted approaches require the clinician's *a priori* knowledge of the potential targets to order the appropriate diagnostic tests.

The application of NGS in metagenomics has revolutionized the field of microbial ecology and greatly facilitates the identification and classification of microbes. The enormous increase in sequencing throughput has enabled the adoption of metagenomic sequencing approaches in which highly complex communities of microorganisms are sequenced in parallel. Compared to the traditional culture-based and assay-based approaches, metagenomic approaches are less biased because they do not require any *a priori* knowledge of the sample composition. Clinical samples may contain a mixture of microbes with varying levels of constituents and additional DNA from a host organism. Metagenomic sequencing data obtained from such samples provides a qualitative and quantitative profile of the individual components of the respective microbial community. Genus, species and even strain-level taxonomic assignments of microorganisms, as well as their relative abundance, could be potentially obtained. For example, metagenomic sequencing data can identify infections with pathogen-specific strain (Maxson and Mitchell, 2016). It also allows the detection and identification of antibiotic resistant genes and virulence factors in complex samples (Jitwasinkul et al., 2016). The ability to rapidly characterize and identify the entire microbial composition of a complex sample provides a unique and novel strategy for pathogen detection and identification in diagnosis and outbreak investigation of infectious diseases, or to guide treatment options.

On the other hand, metagenomic data brings new challenges for downstream analysis and biologically meaningful interpretation. First of all, the vast amount of sequencing data which contains billions of short reads leads to high time consumption. The short read length and low coverage would result in many short contigs and unassembled sequences, leading to the prediction of a large number of small, fragmented genes which may not exhibit any matches in the reference sequence database, or match with low confidence. The second challenge lies in the sample complexity (Rose et al., 2015), as the target pathogens could be surrounded by a complex background of commensal organisms at a range of abundances in addition to hosting nucleic acids. In addition, problems arise from variation between similar subspecies, genomic sequence similarity between different species, the difference in abundance for species in a sample, and different sequencing depths for individual species, etc.

In pathogen identification from metagenome data, strain-level bacterial typing from uncultured food samples is an especially challenging task. Advances in metagenome bioinformatics over the last decade have refined the resolution of microbial community taxonomic profiling from phylum to the species, but it is still challenging to characterize microbes in communities at strain level (Truong et al., 2017). Strain typing distinguishes between different strains of the same species, and is more valuable in a number of specialized fields including epidemiology, compared to species level typing. More specifically, strain typing helps to trace the source of food poisoning and relate individual cases to an outbreak of infectious disease. Strain level variants within microbial species are crucial in determining their functional capacities within the human microbiome (Truong et al., 2017). Strain typing of a single genome has been well studied (Li et al., 2009). However, the tools built under the assumption of assembling a single genome often underperform when used for complex metagenome assemblies. Salmonella is a diverse genus of Gram-negative bacilli and a major foodborne pathogen responsible for more than a million illnesses annually in the United States alone. In particular, strain typing for foodborne pathogen such as *S. enterica* is of special interest and importance (Bell et al., 2016). Methods specific for *Salmonella* detection and identification have been proposed in the literature, including serotyping (Zhang et al., 2015; Yachison et al., 2017), multilocus sequence typing (MLST) (Ranjbar et al., 2017), and strain typing (Hong et al., 2014b; Wood and Salzberg, 2014; Ahn et al., 2015; Truong et al., 2015), etc. However, as different *S. enterica* strains share many common genome regions that are very similar to those from other bacteria in food samples, the accuracy of traditional strain typing methods is not satisfactory especially when the target strain has very low abundance.

In this paper, we introduce UltraStrain, which is a highly sensitive strain typing method based on shot-gun sequencing data. The method exploits the concept of species-specific marker genes (Segata et al., 2012) that are used as genetic proxies of species to efficiently extract high-confidence *S. enterica* reads from the metagenomics sample, whereby subsequent strain typing is performed on a large pool of *S. enterica* reference database based on the high confident *S. enterica* reads. More specifically, in UltraStrain, we first perform a denoise filtering step to remove ambiguous reads that may come from other bacteria or species other than *S. enterica*. This is done by mapping the raw shot-gun sequencing reads to a synthetic reference genome that contains only specific genome regions for *S. enterica*, and keeping only reads that could be successfully mapped to the synthetic reference genome on certain criteria. After that, we compare the resulting high-confidence *S. enterica* specific reads against a pool of known *S. enterica* strains, and formulate strain identification as statistical learning problem, as to identify the probabilities of *S. enterica* strains that could be able to produce those reads if they were present in the original sample. A preliminary version of UltraStrain was used in our submission to PrecisionFDA's CFSAN Pathogen Detection Challenge in 2018 and was one of the top performers in this competition (https://precision.fda.gov/challenges/2/view/results).

## 2. Related Work

Taxonomic profiling of metagenome data can be done by aligning every read to a large database of genomic sequences using BLAST (https://blast.ncbi.nlm.nih.gov/Blast.cgi). However, this is always not clinically applicable due to the large data amount. Other methods for strain typing from metagenome data include *de novo* assembly based methods and mapping based methods. Depending on how the reference sequence library is constructed, mapping based methods further include *k*-mer and marker-gene based methods, and those that map reads to full reference genomes.

Metagenomic assembly of single isolates can be used to identify strains of uncharacterized species with high sensitivity. Strain level metagenomic assembly methods, such as the Lineage (OBrien et al., 2014) and the DESMAN algorithms (Quince et al., 2017), typically use contig binning and statistical analysis of base frequencies across different strains in the sample to resolve ambiguities. The intuition behind is that the frequencies of variants associated with a strain fluctuate with the abundance of that strain. However, metagenomic assembly for multiple strains is computationally challenging. In addition, especially for complex clinical samples when multiple similar strains co-exist, it is generally impossible for assembly based method to achieve high accuracy on strain level due to the conserved regions between strains. Instead, direct assembly of multiple similar strains always produces highly fragmented assemblies which represent aggregates of multiple similar strains. Therefore, it is difficult to generalize assembly-based approaches to large sets of metagenomes and low abundance microbes.

Mapping based methods align the reads to a target reference library and apply statistical and probabilistic analysis techniques on the alignment results to identify the multiple strains that present in the sample. Raw reads of a metagenome can be aligned against full reference genomes for microbe identification if the library of target reference genomes can be constructed. Short read alignment-based methods can achieve high accuracy in strain level identification and are considerably faster than metagenome assembly based methods. Sigma (Ahn et al., 2015) is a read mapping based method that maps the metagenomic dataset onto a user-defined database of reference genomes. A probabilistic model is used to identify and quantify genomes, and the reads are assigned to their most likely reference genomes for variant calling. PathoScope2 (Hong et al., 2014b) builds a complete pipeline for taxonomic profiling and abundance estimation from metagenomic data, integrating modules for reads quality control (Hong et al., 2014a), reference library preparation, filtering of host and non-target reads (Byrd et al., 2014), alignment, and Bayesian statistical inference to estimate the posterior probability profiles of identified organisms (Francis et al., 2013), etc. It can quantify the proportions of reads from individual microbial strains in metagenomic data from environmental or clinical samples.

To speed up the alignment process, the reference library may contain only part of the whole reference genomes that have differentiating power among different but closely related strains. In such methods, metagenomic reads are aligned to a set of preselected marker sequences, e.g., *k*-mers, marker genes, or even pangenomes, and assigned to its most likely origin according to the alignment results. The taxonomic classification can be inferred from phylogenetic distances to these marker sequences. These methods differ in terms of the selection of the markers and the probabilistic algorithms for read assignment. The performance also heavily depends on the completeness of the reference database, and how the marker sequences are extracted.

Kraken (Wood and Salzberg, 2014) is a fast *k*-mer based method for metagenomic sequence classification. Kraken builds a database that contains records consisting of a *k*-mer and the lowest common ancestor (LCA) of all organisms whose genomes contain that *k*-mer. The database is built from a user-specified library of genomes and allows quick look-up of the most specific node in the taxonomic tree, leading to fast and accurate strain identification. StrainSeeker (Roosaare et al., 2017) constructs a list of specific *k*-mers for each node of a given guide tree, whose leaves are all the strains, and analyzes the observed and expected fractions of node-specific *k*-mers to test the presence of each node in the sample. MetaPhlAn (Segata et al., 2012) is a taxonomic profiling method using marker genes. The method estimates the relative abundance of microbial cells by mapping reads against a reduced set of clade-specific marker sequences that unequivocally identify specific microbial clades at the species level and cover all of the main functional categories. MetaPhlAn2 (Truong et al., 2015) further extends the reference library from species level markers to subspecies markers that enable strain-level analysis, and increases the accuracy on taxonomic composition reconstruction. PanPhlAn (Scholz et al., 2016) builds a pangenome of the species of interest by extracting all genes from available reference genomes and merging them into gene family clusters. The method then leverages gene family co-abundance within a metagenomic sample to identify strain-specific gene repertoires, with the assumption that single-copy genes from the same genome should have comparable sequencing coverage within the sample.

## 3. Methods

**Figure 1:**
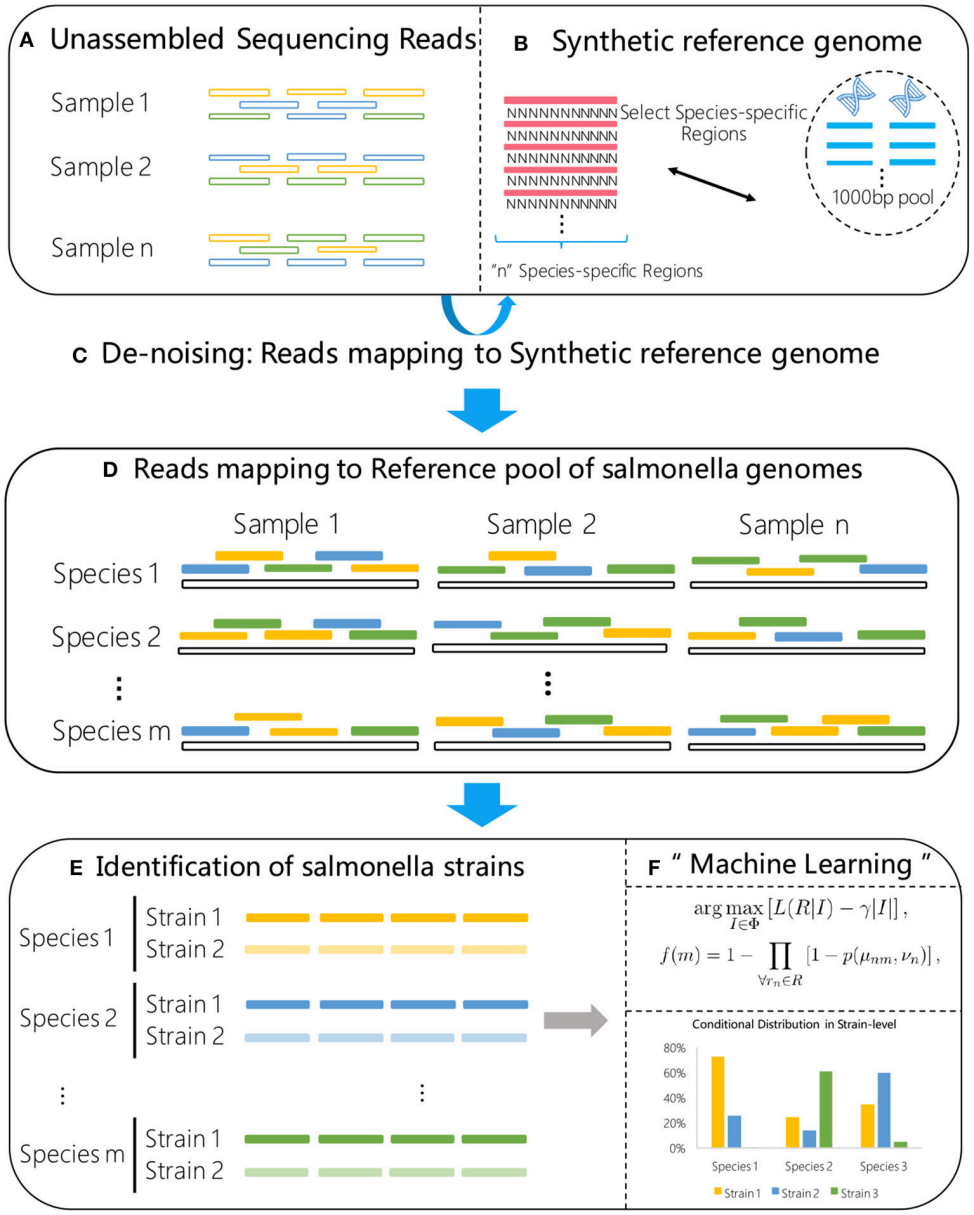
Flow chart of the proposed method. A synthetic reference genome **(B)** is first constructed by concatenating *S. enterica* specific marker sequences, and used to select the high confidence *S. enterica* reads **(C)** from the raw metagenome sample data **(A)**. The selected reads are then aligned to a reference library consisting of known *S. enterica* strains **(D)**. Using the alignment results **(E)** as input, a statistical machine learning algorithm **(F)** is proposed for high sensitive strain identification.

In this paper, we present an ultra sensitive pipeline for *S. enterica* strain typing from metagenomics samples based on NGS data analysis. The processing modules involved in the proposed pipeline are illustrated in Figure 1. The major components of the pipeline include quality control (QC), reads filtering and strain identification.

### 3.1. Quality Control

The first step of the metagenomic sequencing data processing is quality control (QC). The QC procedure usually includes identification and filtration of sequencing artifacts such as low-quality reads and contaminating reads, which would significantly affect and sometimes mislead downstream analysis. In our method, we apply fastp (v.0.19.4; http://opengene.org/fastp/fastp) (Chen et al., 2018) to trim the reads in the front and the tail. For all the raw reads used in our experiments, we trim the front of both reads in a pair with fastp options (-f 15 -F 15), and perform per-read cutting by quality in the tail (–cut_by_quality3).

### 3.2. Reads Filtering

Metagenomics samples could be contaminated with DNA from host genomes or commensal species. Such background noise will often dominate metagenomics samples, which can swamp out target signal, resulting in inaccurate analysis and even leading to incorrect strain identification results. To mitigate this issue, in this step we filter out reads that are not specific to *S. enterica* to minimize potential false positive results in strain identification. This is achieved by aligning the reads after QC to a synthetic reference genome which is composed of *S. enterica* specific regions. Only the properly mapped paired reads that meet certain criteria will be retained for further analysis. The read filtering module consists of the following two steps.

#### 3.2.1. Generating a Synthetic Reference Genome

We follow the method in Laing et al. (2017) to identify species-specific regions for *S. enterica*. First of all, Panseq Laing et al. (2010) is used to identify regions of 1000 bp from closed *S. enterica* genomes in GenBank. These regions are then screened against the online GenBank non-redundant (nr) database to filter out genomic regions that also present in other bacterial genomic sequences. The resulting 403 regions, 1,000 bp each, are identified as marker genomic regions that represent *S. enterica* species.

These regions are concatenated into a single sequence to create a synthetic reference genome that represents the *S. enterica* species. During the concatenation, we insert “separating regions” of repeating N's in-between of the adjacent regions, as shown in Figure 1B. The purpose of inserting such separating regions is to avoid the unfavorable case when a read is mapped to a subsequence on the synthetic reference genome that overlaps with two different *S. enterica* specific regions. The length of the separating regions, or the number of N's, can be set to one more than the maximum read length. In our experiments, we use a large number of 500. The resulting synthetic reference genome is then used to identify reads that can be mapped to unique *S. enterica* genome regions from the shotgun sequencing data for further strain typing.

#### 3.2.2. Read Filtering Through Alignments

We align the sample reads after QC to the synthetic reference genome using BWA (v.0.7.12-r1039; https://github.com/lh3/bwa.git) (Li, 2013). We then analyze the resulting SAM file to filter the reads such that only high confidence *S. enterica* specific reads that are “properly mapped” to the synthetic reference genome are retained.

A read is considered to be “properly mapped” if all the following criteria are met. First of all, its edit distance to the reference genome is no larger than a predefined threshold, with default value of 5 in our implementations. Secondly, the total length of soft clipping bases is no larger than a predefined threshold, with default value 10. Lastly, paired-end reads are retained only if both reads satisfy the above two criteria. The filtering is implemented in Python using the pysam (https://github.com/pysam-developers/pysam) module.

The alignments in the SAM file that pass the filtering are then converted back to fastq format using Picard tools (http://broadinstitute.github.io/picard) as input to the strain identification module.

### 3.3. Strain Identification

#### 3.3.1. Building a Reference Library of *S. enterica* Genomes

A basic step for strain identification from metagenomics sequencing data is to build a library of reference genomes, which contains all the possible strains that may exist in the sample. In this work, we also create a reference genomes library containing known *S. enterica* strains. First, we download all the closed *S. enterica* reference genomes from NCBI. At the time when experiments presented in this paper were performed, we downloaded 380 whole *S. enterica* genomes and 157 chromosomes from NCBI which contain the main sequence and plasmids. We remove the plasmids and keep only the main sequence.

#### 3.3.2. Identification of *S. enterica* Strains

At this stage, we try to identify a subset of *S. enterica* strains from the reference library that best explains the *S. enterica* specific reads present in the sample. The strain identification problem can be formulated as a statistical inference problem that identifies a set of *S. enterica* strains that maximizes the likelihood of the observed *S. enterica* specific reads, as it is unlikely that those reads are from non *S. enterica* strains. Let Φ = {ϕ_*m*_|*m* = 1, …, *M*} denote the reference library where each ϕ_*m*_ represents a known *S. enterica* strain. Let *R* = {*r*_*n*_|*n* = 1, …, *N*} denote the set of high confidence *S. enterica* specific reads after QC and read filtering steps. The strain typing problem can be formulated as:

(1) $$\begin{array}{l} \arg\underset{I\in \Phi }{\max}\left[L\left(R|I\right)-\gamma |I|\right], \end{array}$$

where *L*(*R*|*I*) is the likelihood of *R* under the assumption that a subset of *S. enterica* strains *I* are present in the sample under test, | · | is the cardinality of a set, and γ is a regulator parameter introduced to avoid trivial solutions such as using the entire reference library as the optimal solution. Note that the parameter γ controls the sparsity level of the solution. The larger the value γ is, the fewer potential candidate strains will be included in the solution.

The optimization problem Equation (1) is a minimum set cover problem, which is typically solved using integer linear programming (ILP) (Garfinkel and Nemhauser, 1972). However, the optimal solution of minimum set cover problem is NP-hard and intractable for large data sets. Instead, in this work we propose an alternative statistical learning based method to solve this problem. More specifically, denote *x*_*nm*_ = 1 if a read *r*_*n*_ is from strain ϕ_*m*_, and *x*_*nm*_ = 0 otherwise. We notice that *x*_*nm*_ is a random variable of which the probability distribution by and large depends on how well *r*_*n*_ maps to ϕ_*m*_, and the number of reference genomes in Φ that *r*_*n*_ can be successfully mapped to.

Denote such a conditional probability as *P*(*x*_*nm*_ = 1|μ_*nm*_, ν_*n*_), where μ_*nm*_ is the editing distance from read *r*_*n*_ to reference ϕ_*m*_, and ν_*n*_ denotes the number of reference genomes in the library that read *r*_*n*_ has successfully mapped to. The probability of whether a strain ϕ_*m*_ is present in the sample is given by 1 minus the joint probability of *x*_*nm*_ = 0 for all the reads *r*_*n*_ ∈ *R*, i.e.,

(2) $$\begin{array}{l} f\left(m\right)=1-\underset{\forall r_{n}\in R}{\prod }\left[1-p\left(\mu_{nm},\nu_{n}\right)\right], \end{array}$$

where *p*(μ, ν) ≜ *P*(*x* = 1|μ, ν). In actual implementation, *p*(μ, ν) can be trained from generated metagenomic samples with spike-in reads from known *S. enterica* strains. Once the values for *p*(μ, ν) are trained, for a given sample under test, strain-typing can be accomplished by identifying strains with highest *f*(*m*) calculating using Equation (2) from the alignment information (μ_*nm*_, ν_*n*_) of all the *S. enterica* specific reads from the sample.

#### 3.3.3. Refinement

In our experiments, we observed that for sample with very low *S. enterica* abundance, there could be more than one candidate *S. enterica* strains with highest *f*(*m*) since there are not enough *S. enterica* specific reads to identify the true target strain using Equation (2). To further improve the specificity of the proposed algorithm, in this case an additional reassignment step is conducted where the statistical inference procedure Equation (2) is performed again on a subset of reference library that contains only the top *N* candidate strains obtained from previous step using all the reads from the entire sample after the quality control step. The final candidate strains are identified from the highest probability *f*(*m*) after the refinement step.

## 4. Experimental Results

In this section, we first describe the training of the conditional probability distribution table from simulated training data. Then, we evaluate the sensitivity of the proposed UltraStrain method and compare with three existing methods, namely, Kraken (Wood and Salzberg, 2014), Sigma (Ahn et al., 2015), and Pathoscope2 (Hong et al., 2014b). For all the algorithm test, the same library of *S. enterica* genomes as described in section 3 was used. Simulated metagenome sequencing data, which were created by merging reads from target strains with reads from real background microbial samples at various spike-in levels, were used in performance evaluation as they provide necessary ground truth information. We then further evaluated the performance of the proposed method using data from PrecisionFDA's CFSAN Pathogen Detection Challenge (https://precision.fda.gov/challenges/2). Finally, we compared the runtime performance of these methods using two set of samples generated from dataset of PrecisionFDA CFSAN Pathogen Detection Challenge.

### 4.1. Training of Conditional Probability Distribution Table

First, we created a training data set for the purpose of learning the conditional probability distribution table. The training set included 1,100 simulated samples, which were created using ART simulator (Huang et al., 2011) from various *S. enterica* genomes. All simulated reads were created with 250 bases long with error profile that mimics typical MiSeq v1 sequencing machine (options: “-ss MSv1 -p -l 250 -m 300 -s 10 -na”). The generated simulated reads were then filtered using the synthetic *S. enterica* specific reference to obtain reads that mapped to the *S. enterica* specific regions for constructing the conditional probability distribution table as follows.

The *S. enterica* specific reads *r*_*n*_ obtained from previous step were mapped to the reference library Φ, and a condition matrix *C*_*N*×*M*_ was extracted from the alignment results, where *N* denotes the total number of reads being analyzed and *M* denotes the size of the reference library. Each element of *C* is a 2-tuple *C*_*nm*_ = (μ_*nm*_, ν_*n*_), where μ_*nm*_ is the editing distance from read *r*_*n*_ to reference ϕ_*m*_, and ν_*n*_ denotes the number of reference genomes in the library that read *r*_*n*_ has successfully mapped to. Note that read *r*_*n*_ could map to different reference genomes with different editing distance values. For each read *r*_*n*_, the ground truth label *x*_*nm*_ is also available for all reference strains ϕ_*m*_, i.e., *x*_*nm*_ = 1 if read *r*_*n*_ comes from strain ϕ_*m*_ and *x*_*nm*_ = 0 otherwise.

For each (μ_*nm*_, ν_*n*_)-tuple, we counted the number of occurrences when *x*_*nm*_ = 1 and *x*_*nm*_ = 0, respectively, as follows:

(3) $$c_{\left(\mu_{nm},\nu_{n}\right)}^{+}=\;|\underset{x_{nm}=1}{\cup }\{(\mu_{nm},\nu_{n})\}|$$

(4) $$c_{\left(\mu_{nm},\nu_{n}\right)}^{-}=|\underset{x_{nm}=0}{\cup }\{(\mu_{nm},\nu_{n})\}|.$$

The conditional probability of a positive hit can then be calculated as

(5) $$\begin{array}{l} p\left(\mu_{nm},\nu_{n}\right)=\frac{c_{\left(\mu_{nm},\nu_{n}\right)}^{+}}{c_{\left(\mu_{nm},\nu_{n}\right)}^{+}+c_{\left(\mu_{nm},\nu_{n}\right)}^{-}} \end{array}$$

Due to the large number of strains in the reference library, the total number of possible conditions is large. This may cause the so-called “null context” problem where some conditions may only have very small number of occurrences, leading to inaccurate estimation of probability. This problem can be overcome by reducing the number of conditions using non-uniform binning method on ν_*n*_. Specifically, we grouped values of ν_*n*_ into a number of bins with different sizes. The calculation of conditional probabilities is then performed on the grouped bins using accumulated counting from those of all the ν_*n*_ inside each bin. In our simulation, we used 6 bins which are {[0, 2), [2, 5), [5, 10), [10, 30), [30, 100), [100, ∞)} where the last bin covers all ν_*n*_ values that are not less than 100.

The learned conditional probability table was then used in the following experiments for strain identification by calculating the probability of presence of each candidate strain from the library as described in section 3.

### 4.2. Experiment on Abundance

To evaluate the performance of the proposed UltraStrain, we generated 65 synthetic sample data with spike-in of different *S. enterica* strains at different abundance levels for testing. The background reads in the synthetic samples were produced from a mixture of simulated reads generated from 10 non *S. enterica* genomes listed in Table 1, and the foreground reads were simulated from 13 target *S. enterica* genomes as listed in Table 2. In both cases the simulated reads were generated using ART read simulator (Huang et al., 2011) with the same parameters as in section 4.1. For the background, the reads were generated at 10x coverage from the 10 listed non *S. enterica* genomes, respectively. In addition, to avoid potential contamination from the background sample, reads that could be aligned to the synthetic *S. enterica* specific reference genome at high quality were removed. Finally, the foreground reads were randomly down-sampled to 5 different abundance levels of 10%, 1%, 0.1%, 0.01%, 0.001% according to the total read number in the background sample, and mixed with the background sample to generate the synthetic testing samples.

**Table 1:** The 10 *non-S. enterica* genomes used as background strains in the simulated data sets.

**Species**	**Strain**	**Taxid**	**ASM name**
*Escherichia coli*	UTI89	364106	ASM1326v1
*Shewanella putrefaciens*	97	24	ASM331542v1DOE
*Campylobacter fetus* subsp. *testudinum*	D6856	1507806	ASM169948v1
*Campylobacter jejuni*	OXC6265	197	7038_3_16
*Borreliella burgdorferi*	IPT92	1408876	BorBurgIPT92
*Campylobacter coli*	BIGS0010	1247735	ASM31420v1
*Helicobacter pylori*	NAB47	1156914	ASM25607v2
*Leptospira interrogans serovar Copenhageni*	HAI0156	996862	CLC_glsol191
*Buchnera aphidicola*	LL01	713603	ASM18322v1
*Azorhizobium caulinodans*	ORS 571	438753	ASM1052v1

**Table 2:** The 13 *S. enterica* genomes used as target strains in the simulated data sets.

**Species**	**Strain**	**Taxid**	**ASM name**	**Genbank accession**
Albany	ATCC 51960	1173798	ASM48751v2	CP019177.1
Choleraesuis	SCB67	321314	ASM810v1	AE017220.1
Enteritidis	EC20121178	1412595	ASM62309v2	CP007271.2
Heidelberg	SH14009	611	ASM169265v1	CP016581.1
Infantis	N55391	595	ASM193159v1	CP016410.1
Newport	0307213	108619	ASM127831v1	CP012599.1
Paratyphi A	ATCC 9150	295319	ASM1188v1	CP000026.1
Pullorum	ATCC 9120	1029979	ASM33048v2	CP012347.1
Saintpaul	SARA26	702982	ASM48616v2	CP017727.1
Typhi	CT18	220341	ASM19599v1	AL513382.1
Typhimurium	SO2	28901	ASM157627v1	CP014356.1
Montevideo	USDAARSUSMARC1903	1454603	ASM94097v1	CP007222.1
Anatum	CDC 060532	1454592	ASM94089v2	CP007271.2

**Figure 2:**
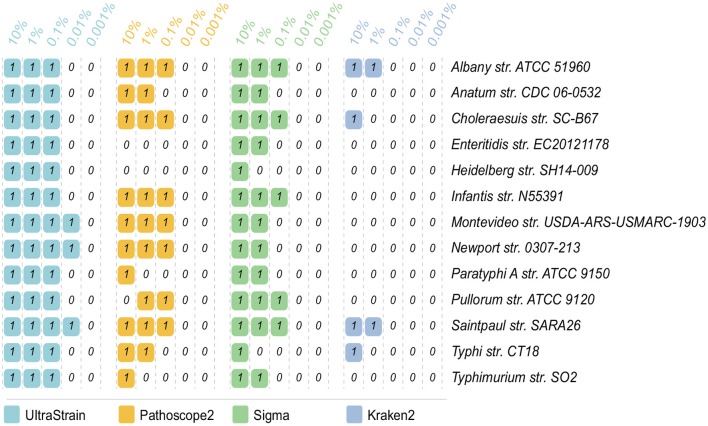
Comparison of UltraStrain, Pathoscope2, Sigma, and Kraken2 in strain identification from 65 simulated data sets. The reads from each of the 13 target strains, as listed in the rightmost column, are mixed with the reads from the background strains, at 5 different abundance levels from 10% to 0.001%. “1” means that the method successfully identifies the target strain from the simulated sample data, while “0” means failure, i.e., the method either identifies a different *S. enterica* strain as the most probable strain, or did not identify any *S. enterica* strains from the simulated sample data.

The strain identification results on the 65 data sets for the abundance test are showed in Figure 2. In can be seen from the results that UltraStrain perform best in correctly identifying the target strains. In particular, UltraStrain correctly identifies all the 13 strains at 0.1%, while Pathoscope2, Sigma, and Kraken2 only correctly identify 7, 5, and 0 strains, respectively. In addition, UltraStrain could still correctly identifies 4 out of 13 strains at 0.01% abundance while all the other algorithms under test failed to identify the correct strain at this abundance level.

### 4.3. Experiments on Coverage

It is interesting to note that due to the filtering process used in the algorithm, the sensitivity of UltraStrain will be increased if more metagenomic data are available for a given sample. That is, for a given sample with low abundance of *S. enterica* contamination, the chance of UltraStrain to correctly identify its strain will be higher if it is sequenced to higher coverage. This is because that with higher coverage of data, more *S. enterica* specific reads will be retained after the filtering operation. Hence it will give better chance for UltraStrain to correctly identify the target strain. Note that this property is in general not applicable to other strain typing software since the ratio of reads from *S. enterica* vs. other species simultaneously present in the sample will remain constant without the filtering operation.

**Figure 3:**
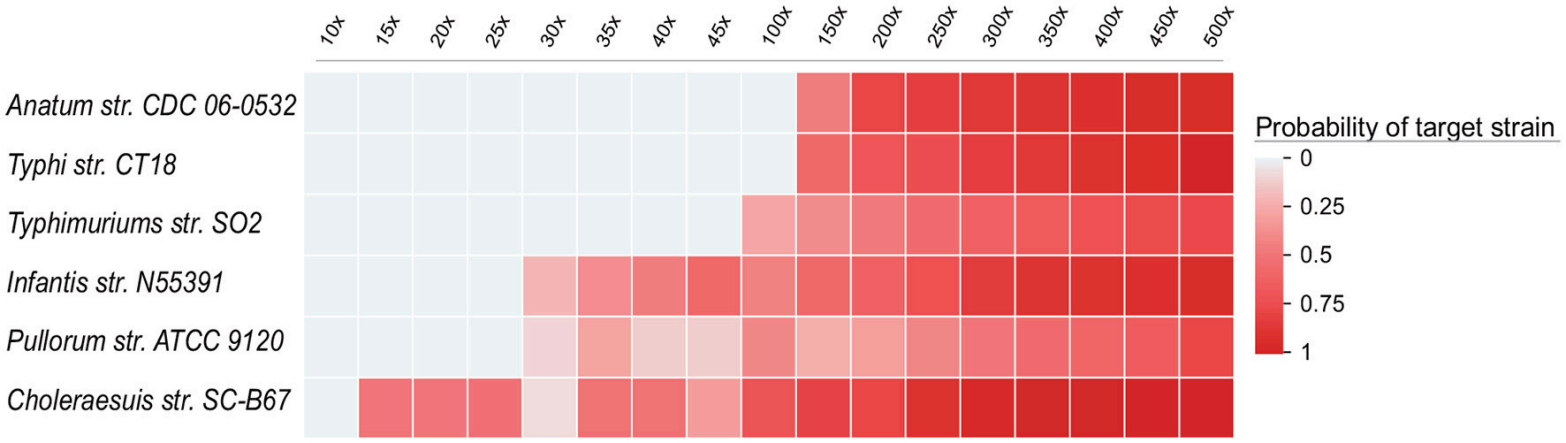
Performance of UltraStrain on the 102 simulated data sets in the coverage experiments. In all the data sets, the abundance level of the target strain is fixed at 0.01%, while the coverage ranges from 10× to 500×. The probability of the target strain keep increasing with increasing coverage. At a higher coverage, UltraStrain is able to correctly identify the target strain that it cannot detect at lower coverage. The other three algorithms (Pathoscope2, Sigma and Kraken2) were not able to identify the correct strains under all testing conditions.

To illustrate that the sensitivity of UltraStrain will be increased with higher coverage data, we further evaluated the performance of UltraStrain on metagenomic data of different coverage. The same procedure in previous sector was followed to create the testing data. The synthetic background reads were generated from 10 non *S. enterica* strains at 17 different coverage values ranging from 10×, 15×, ⋯ , to 500×, and the target *S. enterica* reads were spiked-in at constant abundance level of 0.01%. In total, 102 test data sets were generated for this experiment. Figure 3 shows the performance of UltraStrain on the testing data. It can be seen that with increasing coverage, the calculated probability of target strain is also increased. Note that the increment is not monotonically due to the randomness nature of the number of spiked-in reads present in the *S. enterica* specific genome region. However, at higher coverage, UltraStrain is able to correctly identify the target that it is not able to detect at lower coverage.

We had also tested other three algorithms (Pathoscope2, Sigma, and Kraken2). However, none of them was able to correctly identify the target strain under all testing conditions.

### 4.4. Results on FDA CFSAN Pathogen Detection Challenge

The PrecisionFDA CFSAN Pathogen Detection Challenge (https://precision.fda.gov/challenges/2/) aims at detecting *S. enterica* in shotgun metagenomic samples from contaminated cilantro. The goal of the challenge was to identify and type *Salmonella* in naturally and *in silico* contaminated samples. The Challenge provided 24 test samples, and the participants were asked to identify the serotype, sequence type (i.e., MLST), and strain of *Salmonella* present in positive challenge samples.

**Figure 4:**
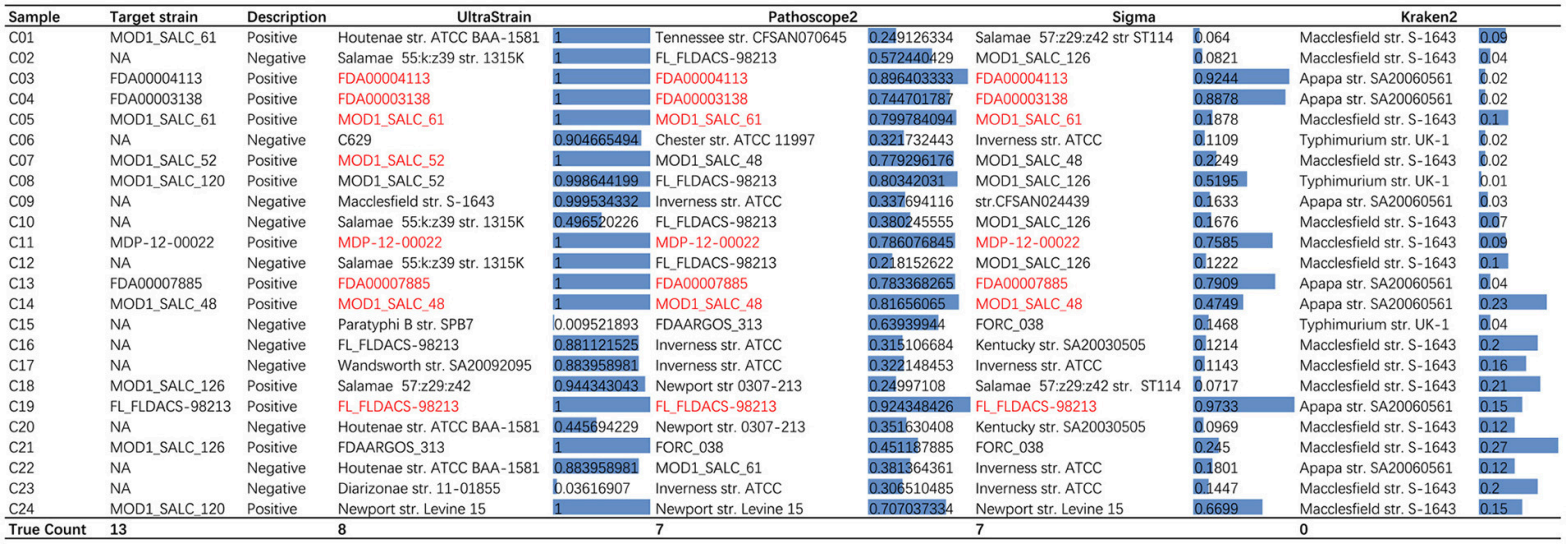
Comparison of performance of UltraStrain, Pathoscope2, Sigma, and Kraken2 on PrecisionFDA CFSAN Pathogen Detection Challenge data set. For each testing sample, the most probable strains identified by the algorithms are shown. Correctly identified strains are marked with red color. For UltraStrain, Pathoscope2, and Sigma, the scores reported in the figure indicate the probabilities of the identified strains present in the sample. For Kraken2, the scores indicate the related abundances of the identified strains.

We tested the performance of UltraStrain on the 24 challenge samples, and the results are shown in Figure 4. Among these 24 samples, 13 are positive, including 5 *in silico* synthetic samples with a spike-in known *S. enterica* target strain into the culture-negative samples, and 8 culture-positive samples. The remaining 11 samples are culture-negative samples. UltraStrain correctly identified the target *S. enterica* strain in 8 positive samples (5 *in silico* and 3 culture-positive samples). Both Pathoscope2 and Sigma successfully identified the target strain in 7 samples, while Kraken failed in all samples. However, for culture-positive samples C01, C08, C18, C21, and C24, none of the four methods can identify the correct *S. enterica* strain.

It can be seen from the results that for some negative samples, UltraStrain still identify target strains with very high probabilities. This could possibly be due to two reasons. First, the negative sample may not be truly negative due to the high sensitivity of UltraStrain. In particular, there are still some amount of *S. enterica* specific reads left after the filtering process, which may suggest that the sample may contain certain level of *S. enterica* contamination. Secondly, it is possible that the sensitivity of UltraStrain could be too high for real-life samples. Therefore, it is possible that we select a higher cut-off value of probability (e.g., 0.99) when it is used for *S. enterica* detection.

### 4.5. Experiments on Runtime

**Figure 5:**
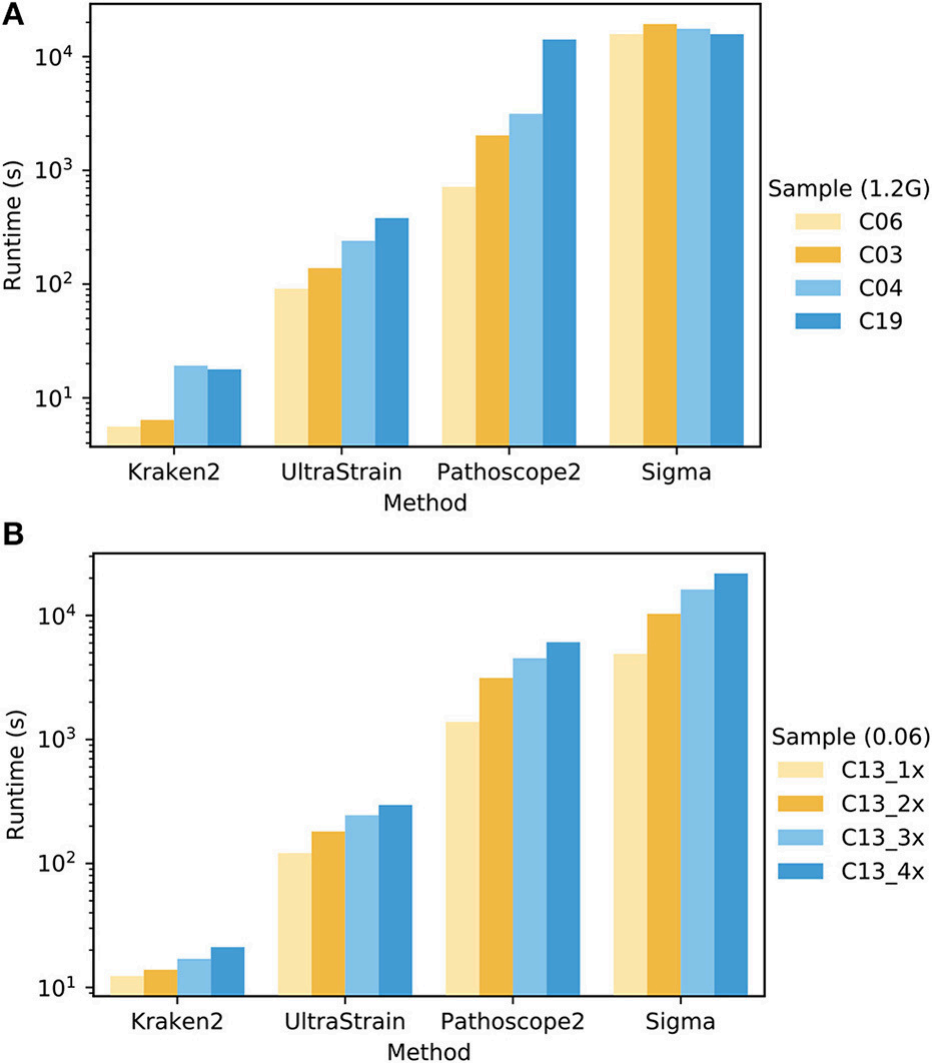
Comparison of the runtime performance of UltraStrain, Pathoscope2, Sigma and Kraken2. **(A)**. Performance on four different samples (C03, C04, C06, C19 from PrecisionFDA CFSAN challenge) that have different levels of *S. enterica* abundance of 0.00 (C06), 0.005 (C03), 0.03 (C04), 0.06 (C19). For fair comparison, all the files are truncated to 1.2 GB. **(B)** Performance on four samples with increasing file size from 318 MB (1×) to 1.3 GB (4×) constructed by duplicating testing sample C13. A higher bar indicates a computationally more expensive process. Note that the runtime results are shown in logarithmic scale.

To compare the computational complexity of UltraStrain in terms of runtime with other methods, we tested the runtime performance of all four methods using two sets of samples selected from PrecisionFDA CFSAN challenge dataset. The experiments were conducted on an Intel Xeon workstation with 48 CPU threads and 256 GB RAM. All methods were run with their default settings, and set to utilize up to 44 CPU threads whenever it is possible. The results are shown in Figure 5. It can be seen that the runtime performance of these tools varies dramatically, which can take from 10^1^ to 10^4^ seconds per test depending on respective method as well as the sizes and compositions of samples under test. In general, the runtime of each tool increases as the file sizes of testing samples increase. In addition, the runtimes of UltraStrain and Pathoscope2 also increase as the abundances of the target spike-in strains increase, which is reasonable as there will be more matched reads to be processed in both algorithms when the abundances of target strains increase. Overall, Kraken2 has lowest complexity among all tools. UltraStrain has the second lowest complexity followed by Pathoscope2. Sigma has the highest complexity in all cases.

## 5. Conclusions

UltraStrain is a highly sensitive, rapid and efficient method for metagenomic taxonomic classification at strain level. In UltraStrain pipeline, the reads filtering step uses a synthetic reference genome consisting of differentiating regions from known *S. enterica* strains to filter out the reads that are not specific to *S. enterica* species, greatly improving the efficacy as well as efficiency of the process. Strain identification through the proposed statistical learning provides a fast and accurate solution for metagenome sample data analysis. Experiments on both simulated data sets and real sample demonstrate that UltraStrain achieves high accuracy even at very low abundance level. Ultrastrain achieves both shorter run time and higher sensitivity, which indicates its usability as a standalone pathogen identification pipeline. In addition, our experiments show that the sensitivity of UltraStrain can be further improved by using deeper sequencing of the sample, which could be particularly useful when it is necessary to perform strain typing on sample with extremely low abundance of target strains.

The proposed algorithm can be further improved in many aspects. For example, although it is developed with the target of high-sensitivity *S. enterica* in mind, the proposed framework can be easily extended to taxonomic profiling and analyze other bacteria strains by adapting its filter and reference library designs. In addition, the ability of current algorithm in dealing with sample with more than one target strains from the same species still needs further investigation. Importantly, the current approach, as its primary goal is for ultra sensitive strain typing, lacks the ability to accurately identify the relative abundance of multiple bacteria species/strains present in a sample as provided by other similar tools. Therefore, it is anticipated that it could be used in conjunction with other metagenomic pipelines when necessary.

## Data Availability

Publicly available datasets were analyzed in this study. This data can be found here: https://precision.fda.gov/challenges/2/view.

## Author Contributions

WY and RY: conceptualized the algorithm design and interpreted the data. WY, LW, and RY: designed the study. WY, LH, CS, LW, and RY: collected the data. LH, CS, WY, and RY: analyzed the data. WY, LH, CS, LW, and RY: sourced the literature. WY, LH, LW, and RY: wrote the draft. WY, LW, and RY: edited the manuscript. LW and RY: acquired the funding and supervised the whole study.

### Conflict of Interest Statement

The authors declare that the research was conducted in the absence of any commercial or financial relationships that could be construed as a potential conflict of interest.
